# Lack of evidence of acute HEV infections as a sexually transmitted disease: Data from a German cohort of PrEP users

**DOI:** 10.1016/j.bjid.2024.103720

**Published:** 2024-02-13

**Authors:** Guido Schäfer, Rabea Lübke, Olaf Degen, Maria Mader, Robin Scheiter, Annika Wolski, Marylyn M. Addo, Julian Schulze zur Wiesch, Sven Pischke

**Affiliations:** a ICH Hamburg, Hamburg, Germany; b Ambulanzentrum des UKE, University Medical Center Hamburg-Eppendorf, Hamburg, Germany; c First Department of Medicine, University Medical Center Hamburg-Eppendorf, Hamburg, Germany; d German Center for Infection Research (DZIF), Hamburg-Lübeck-Borstel and Heidelberg Partner sites, Hamburg, Germany

**Keywords:** Hepatitis E, Hepatitis E virus, Pre-exposure prophylaxis, HIV, Seroprevalence STD

## Abstract

**Background:**

While the sexual transmissibility of HAV in MSM has been extensively described, the potential for sexual transmission of HEV has not been definitively established. Although HEV has been detected in the ejaculate of chronically infected men, studies among MSM PrEP users in France did not observe an elevated anti-HEV seroprevalence as an indicator of increased exposure risk by sexual intercourse.

**Patients and methods:**

A total of 111 unselected PrEP users and 111 age- and sex-matched blood donors were tested for anti-HEV IgG, IgM and HEV (PCR). Of the participants 79/111 (71 %) responded to a questionnaire covering topics as sexual preferences, previous sexually transmitted diseases, profession, food consumption, and pet ownership.

**Results:**

The anti-HEV IgG seroprevalence in PrEP users (22 %) did not differ significantly from the rate in controls (17 %). While one PrEP user and three controls tested positive for anti-HEV IgM, all PrEP users and controls tested PCR negative.

**Conclusion:**

In immunocompetent individuals with frequent changes of sexual partners, the epidemiology of Hepatitis E Virus does not significantly involve the sexual transmission route.

## Introduction

Infections with the Hepatitis E Virus (HEV) in the tropics are mostly transmitted through contaminated drinking water and are caused by genotypes 1 and 2.^1^ In contrast, zoonotic infections in industrialized countries are caused by genotypes 3 and 4.^1^ While HEV genotype 1 and 2 infections are always acute and never chronic, HEV genotype 3 and 4 infections can lead to chronic hepatitis E in about 50 % of immunosuppressed patients with acute HEV infections.^2^,^3^ Chronic HEV infections have been defined as virus persistence for longer than 3-months.^3^ In Germany, hepatitis E is a notifiable disease. Less than 4000 cases are annually reported to the Robert-Koch-Institute (www.rki.de).

While hepatitis B and C virus (HBV, HCV) infections are sexually transmissible^4^ and the sexual transmissibility of Hepatitis A Virus (HAV) infections is relevant in the Men who have Sex with Men (MSM) community,^5^ the significance of the possibility of sexual transmissibility for HEV has not yet been conclusively clarified. HBV or HCV infections can be transmitted by vaginal or anal sexual contact.^4^ In contrast, anal and oral sexual contacts are at the forefront regarding sexual HAV transmission.^5^ Like HAV, HEV is excreted in the stool, but differs from HBV or HCV in this regard. Consequently, the question of whether HEV is also sexually transmissible, particularly in MSM, arises naturally. Recently, a study investigated the significance of HEV excretion via ejaculate.^6^ Surprisingly, it was found that the HEV virus concentration in the ejaculate of immunosuppressed individuals with chronic HEV infection can be several log levels higher than the virus load in their blood. In contrast, in the ejaculate of immunocompetent individuals with acute HEV infection, no HEV particles could be detected by PCR. Moreover, HEV was detected in the ejaculate of naturally infected wild boars but not in the testis of experimentally infected pigs.^6^ The implications of these findings still need to be determined.

To evauate the possibility of sexual transmission a study from Toulouse, France, tested 147 MSM receiving Pre-Exposure Prophylaxis (PrEP) against HIV for anti-HEV IgG and IgM.^7^ PreP users form an ideal cohort for investigating potential sexual HEV transmission since PrEP usage can be indicative of engaging in condomless sexual intercourse with multiple partners.

In the Toulouse area, known for its high HEV endemicity, 42 % of MSM tested positive for anti-HEV IgG antibodies, which closely aligns with the 44 % rate among blood donors in this region.^7^ Notably, in this study there was no association between anti-HEV IgG positivity, prior HBV or HCV infections, or a history of sexually transmitted diseases, except for a higher anti HEV seroprevalence among patients with a positive treponema test (56.1% vs. 33.3 %, *p* < 0.01).^7^ At first glance, this study may suggest no link between HEV exposure or anti-HEV positivity and homosexuality with frequent partner changes. Nevertheless, considering the seroprevalence of 42‒44 % in the Toulouse area, the study's discriminatory power for such inquiries may not be sufficient and the findings should be validated in an area with lower HEV endemicity.

Recently another French/Canadian study investigated the anti-HEV seroprevalence in a cohort of 417 MSM PrEP users. Among them, 15 % (*n* = 62) tested positive for anti-HEV IgG.^8^ This study found an association between anti-HEV IgG positivity and older age but not with geographical origin, use of recreational drugs, number of sexual partners, or serological status for HAV or bacterial STDs.

In contrast to these previous reports, we investigated the anti-HEV IgG seroprevalence in a well-defined German cohort of PrEP users and compared it with an age- and sex-matched cohort of healthy blood donors. Additionally, study participants in our prospective analysis completed a questionnaire regarding nutrition, profession, or leisure time activities, allowing us to examine the potential influence of these factors. Furthermore, the history of previous sexually transmitted diseases was analyzed in association with anti-HEV IgG status. Patient questionnaires, serology results and patient files were evaluated for this purpose.

## Material and methods

Consecutive PrEP users from our outpatient clinic at the University Medical Center Hamburg-Eppendorf between October 2020 and May 2021 were invited to participate in this prospective investigation. All participants provided written informed consent and the study was approved by the local Ethics Committee of the Medical Council of Hamburg (PV7049). Each patient received a questionnaire (see supplementary data) and voluntarily completed it either in printed form on paper or electronically via smartphone and internet (REDCap, Nashville, USA).

In adherence to the regulatory conditions in Germany, only oral PrEP, specifically the combination of Emtricitabine and Tenofovir Disoproxil Fumarate, is approved for use. Notably, there were no exceptions to this standard within the studied cohort, and injectable PrEP was not employed.

All pertinent medical information, encompassing comprehensive laboratory data (including but not limited to complete blood counts, renal function markers, liver function tests, and results of tests for sexually transmitted infections), was methodically collected, documented, and transferred into a secure and structured database (Redcap). This meticulous data collection approach aimed to provide a holistic understanding of the participants' health status and facilitate a comprehensive analysis of the association between PrEP use and previous HEV exposure within a cohort f immunocompetent individual with frequent changes of sexual partners.

Continuous variables with a non-normal distribution were presented as the median and Interquartile Range (IQR) and compared using the Mann–Whitney *U test*. Categorical variables were presented as numbers (%) and compared using Fisher's exact test. Statistical analyses were conducted using SPSS, version 21.0 (IBM Corp., Armonk, NY, USA).

## Results

111 male PrEP users from our outpatient clinic and 111 age-and sex-matched healthy controls (blood donors) were included in the study. The age ranged from 18 to 65 years (mean years, Stdv 9-years). Among the PrEP users, 24 (22 %) tested positive for anti-HEV IgG, and 1 (1 %) was positive for anti-HEV IgM. In the control cohort 19 (17 %) tested positive for anti-HEV IgG, and 3 were positive for anti-HEV IgM (both: *p* > 0.5). No patient tested positive for HEV by PCR. The PrEP user who tested positive for anti-HEV IgM did not show any signs of hepatitis and lost anti-HEV IgM positivity within 4 months.

**Fig. 1:**
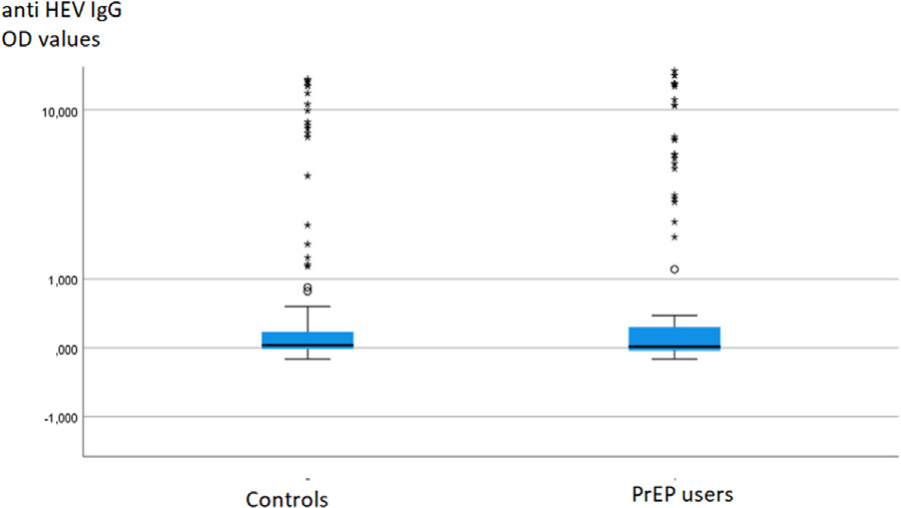
Anti-HEV IgG titers in PrEP users and matched-pair blood donors.

To investigate potential differences in anti-HEV IgG or IgM levels between groups, the data were compared using the Mann-Whitney test, which did not reveal any significant difference (Fig. 1).

A total of 79 out of 111 PrEP users (71 %) responded to the questionnaire regarding sexual preferences, profession, food consumption, and pet ownership (Table 1). Among these respondents, 70 individuals identified themselves as gay (*n* = 60) or bisexual (*n* = 10), 3 as queer, and 6 did not respond regarding their sexual orientation. Additionally, 17 patients reported being in a stable partnership, while 55 denied having one, and 7 did not provide an answer to this question. Among those with a partner, 16/17 (92 %) stated having additional sexual contact partners.

**Table 1:** Responses to the questionnaire for anti-HEV IgG positive and negative PrEP users^a^.

	IgG positive 14/79 (18 %)		IgG negative 65/79 (82 %)	
**PrEP**				
PrEP used	9	64 %	42	65 %
Current prep use	8	57 %	40	61 %
Daily oral prep use	8	57 %	35	54 %
Event-driven prep use	0	0 %	4	6 %
**Residence**				
Hamburg	12	86 %	53	82 %
**Sexuality**				
Homosexual	12	86 %	57	88 %
Queer	0	0 %	2	3 %
Bisexual	1	7 %	0	0 %
**Relationship status**				
Single	12	86 %	42	65 %
In relationship	1	7 %	16	25 %
**Education (highest degree)**				
High school education (“Abitur”)	2	14 %	12	19 %
Main or secondary school (“Haupt-/Real-Schule”)	1	7 %	3	5 %
Apprentice	2	14 %	10	15 %
University	8	57 %	33	51 %
Thesis	0	0 %	1	2 %
**Age**				
< 30	4	29 %	20	31 %
30‒55	8	57 %	39	60 %
> 55	1	7 %	3	5 %
**Country of origin**				
Germany	13	93 %	49	75 %
**Previous Sexual Diseases**				
Had sexual disease	6	43 %	36	55 %
Chlamydia	2	14 %	20	31 %
Gonorrhea	2	14 %	17	26 %
Lues	2	14 %	9	14 %
HAV	0	0 %	0	0 %
HBV	0	0 %	0	0 %
HCV	1	7 %	1	2 %
Herpes	0	0 %	3	5 %
Scabies	2	14 %	10	15 %
Condylomata (HPV)	1	7 %	15	23 %
**Sex partners in the last 6-months**				
More than 9	6	43 %	22	34 %
More than 5	6	43 %	39	60 %
More than 2	9	64 %	54	83 %
**Group sex**				
Once a month	3	21 %	7	11 %
Once a year	2	14 %	7	11 %
Rarely/never	4	29 %	29	45 %
**Vaccination**				
HAV	12	86 %	47	72 %
HBV	12	86 %	48	74 %
**Smoking**				
Never	5	36 %	24	37 %
Formerly	5	36 %	15	23 %
Regulary	3	21 %	13	20 %
Occasionally	0	0 %	6	9 %
**Occupational contact with**				
Pigs	0	0 %	1	2 %
Deer	0	0 %	2	3 %
Sheep	0	0 %	0	0 %
Fruit or vegetables	0	0 %	1	2 %
Sewage	0	0 %	2	3 %
Patients (medical staff)	3	21 %	5	8 %
Fishing	0	0 %	0	0 %
Horse riding	1	7 %	2	3 %
**Travelling outside of Europe (last 6-months)**	6	43 %	41	63 %
**Previous Blood Transfusions**				
Had a blood transfusion	2	14 %	2	3 %
**Consumption of pork**				
Never	0	0 %	4	6 %
1‒5 times a year	3	21 %	4	6 %
6‒2 times a year	2	14 %	15	23 %
> Once a month	9	64 %	41	63 %
**Consumption of horse meat**				
Never	13	93 %	54	83 %
Occasionally	1	7 %	11	17 %
**Consumption of mutton (sheep meat)**				
Never	4	28 %	31	48 %
Occasionally	10	71 %	33	51 %
**Consumption of venison (game meat)**				
Never	5	36 %	28	43 %
Occasionally	9	64 %	37	57 %
**Consumption of cooked shellfish**				
Never	4	28 %	20	31 %
Occasionally	10	71 %	45	69 %
**Consumption of raw shellfish (oysters)**				
Never	10	71 %	46	71 %
Occasionally	4	28 %	19	7 %
**Consumption of sushi**				
Never	5	36 %	16	10 %
Occasionally	9	64 %	16	10 %
**Consumption of spinach/rucola**				
Never	2	14 %	3	5 %
Occasionally	12	86 %	62	95 %
**Consumption of strawberries**				
Never	1	7 %	4	6 %
Occasionally	13	93 %	61	94 %
**Pets**				
Dog	3	21 %	6	9 %
Cat	0	0 %	7	11 %
Fish	1	7 %	0	0 %
Horse	1	7 %	0	0 %
**Frequency of alcohol consumption**				
Less than once a week	3	21 %	16	25 %
Once a week	7	50 %	36	55 %
Daily	1	7 %	4	6 %
**Consumption of tap water**				
Occasionally	2	14 %	9	14 %
< 1 Liter per day	1	7 %	10	15 %
> 1 Liter per day	10	71 %	40	62 %

a Totals do not add up to 100 % in some cases (rounding errors and discrete deviations due to missing answers to individual categories).

Regarding sexual preferences, nor profession, and pet ownership, no statistically significant differences were observed between anti-HEV IgG positive and negative individuals.

Furthermore, we provide a compilation of Sexually Transmitted Infections (STIs) reported in the medical history as well as those that occurred during the observational period (Table 1). Clinical overt acute hepatitis was not observed. Furthermore, no significant elevations in liver enzyme levels were observed within the cohort during the observational period in any of the groups. Overall, sexually transmitted diseases were frequently documented, affecting nearly 50 % of the study population in both groups (Table 1).

## Discussion

The present study clearly demonstrates that there is no significantly elevated anti-HEV seroprevalence in MSM PrEP users compared to sex- and age-matched controls in Germany. Additionally, the anti-HEV IgG titers in PrEP users and controls did not exhibit significant differences (Fig. 1). This observation challenges the assumption of HEV as a sexually transmitted disease in immunocompetent individuals with frequent partner changes engaging in anal sex. Our findings align with previous studies from an area in France with a higher HEV endemicity.

The questionnaire survey conducted as part of our study revealed no significant associations between increased anti-HEV IgG positivity and previous HEV exposure among PrEP users. Notably, partner change frequency and participation in group sex did not correlate with an increased risk of HEV in this group of PrEP users engaging in condomless sex.

Crucially, our study contributes to the growing body of evidence suggesting that sexual transmission of HEV does not play a significant role in MSM in Germany. The detection of HEV particles in the ejaculate of immunocompromised patients with chronic hepatitis E, as opposed to their absence in immunocompetent patients, further supports this observation.

Regarding to the general epidemiology of Hepatitis E in Germany and Europe, it is noteworthy that the perception of Hepatitis E has evolved over the past decade. Previously considered a rare disease in Germany, primarily acquired during travel to Africa or Asia, Hepatitis E is now recognized as a common, foodborne zoonosis dominated by genotypes 3 and 4. This shift is reflected in the reported cases under the German Infection Protection Act (IfSG), where the number of clinically and laboratory-confirmed cases has risen significantly from approximately 20‒30 cases per year in the early 2000s to over 3700 cases in 2019, with the majority lacking travel history (www.rki.de).

It is crucial to note that in contrast to sexual intercourse transient viremias associated with asymptomatic infections in blood donors pose a relevant risk for HEV transmission in Germany, as 1/800 blood donors is HEV viraemic.^9^ As a proactive measure, all donations used for the production of non-virus-inactivated blood products have been screened for HEV RNA since 2020. While contaminated drinking water (genotypes 1 and 2), foodborne/zoonotic transmission (genotypes 3 and 4) and blood transfusions are therefore possible transmission routes for HEV infections, the current study thus makes it clear that sexual transmission does not play a relevant role.

In summary, our study contributes valuable insights by challenging the assumption of HEV as a sexually transmitted disease in a specific population. The broader discussion underscores the changing epidemiology of Hepatitis E in Germany and Europe, emphasizing the need for ongoing surveillance and interventions to mitigate potential risks associated with foodborne transmission. Together with the previous studies from France, there is now sufficient evidence to assert that patients with acute hepatitis E can engage in sexual intercourse safely. This observation is of significant importance since based on these data, there is no reason for patients with hepatitis E to abstain from unprotected sexual intercourse. However, the situation for immunosuppressed patients with chronic hepatitis E has not been definitively clarified yet.

## Author's contribution

G. Schäfer and R. Lübke: These authors contributed equally and share the first authorship.

J. Schulze zur Wiesch and S. Pischke: These authors contributed equally and share the senior authorship.

This work formed part of the thesis of R. Lübcke.

## Conflicts of interest

The authors declare no conflicts of interest.
